# Inhibitory synaptic transmissions to the bed nucleus of the stria terminalis neurons projecting to the ventral tegmental area are enhanced in rats exposed to chronic mild stress

**DOI:** 10.1186/s13041-020-00684-4

**Published:** 2020-10-15

**Authors:** Ryuto Hara, Daiki Takahashi, Tatsuhiro Takehara, Taiju Amano, Masabumi Minami

**Affiliations:** Department of Pharmacology, Graduate School of Pharmaceutical Sciences, Hokkaido University, Sapporo, 060-0812 Japan

**Keywords:** Bed nucleus of the stria terminalis, Chronic mild stress, Chronic pain, Depression

## Abstract

The comorbidities of depression and chronic pain have long been recognized in the clinic, and several preclinical studies have demonstrated depression-like behaviors in animal models of chronic pain. These findings suggest a common neuronal basis for depression and chronic pain. Recently, we reported that the mesolimbic dopaminergic system was tonically suppressed during chronic pain by enhanced inhibitory synaptic inputs to neurons projecting from the dorsolateral bed nucleus of the stria terminalis (dlBNST) to the ventral tegmental area (VTA), suggesting that tonic suppression of the mesolimbic dopaminergic system by this neuroplastic change may be involved in chronic pain-induced depression-like behaviors. In this study, we hypothesized that inhibitory synaptic inputs to VTA-projecting dlBNST neurons are also enhanced in animal models of depression, thereby suppressing the mesolimbic dopaminergic system. To test this hypothesis, we performed whole-cell patch-clamp electrophysiology using brain slices prepared from rats exposed to chronic mild stress (CMS), a widely used animal model of depression. The results showed a significant enhancement in the frequency of spontaneous inhibitory postsynaptic currents in VTA-projecting dlBNST neurons in the CMS group compared with the no stress group. The findings revealed enhanced inhibitory synaptic inputs to VTA-projecting dlBNST neurons in this rat model of depression, suggesting that this neuroplastic change is a neuronal mechanism common to depression and chronic pain that causes dysfunction of the mesolimbic dopaminergic system, thereby inducing depression-like behaviors.

## Introduction

The comorbidities of depression and chronic pain have long been recognized in the clinic [1], and several preclinical studies have shown depression-like behaviors in animal models of chronic pain [2]. These findings suggest a common neuronal basis for depression and chronic pain. The mesolimbic dopaminergic pathway from the ventral tegmental area (VTA) to the nucleus accumbens (NAc) is a key player in the mesolimbic reward circuit, and dysfunction in this pathway has been implicated in depression [3]. We previously reported that reward-induced dopamine release in the NAc was suppressed in rats exposed to chronic mild stress (CMS) model [4], a widely used animal model of depression [5]. Dysfunction of the mesolimbic dopaminergic pathway has also been implicated in chronic pain. We demonstrated that reward-induced dopamine release in the NAc [6] and reward-seeking behaviors [7] were suppressed in rats with chronic pain. These findings suggest that suppression of the mesolimbic reward circuit may be a common neuroplastic change underlying depression and chronic pain. Recently, we showed that the mesolimbic dopaminergic system was tonically suppressed during chronic pain by enhanced inhibitory synaptic inputs to neurons projecting from the dorsolateral bed nucleus of the stria terminalis (dlBNST) to the VTA [8]. We previously reported that most VTA-projecting BNST neurons are GABAergic neurons that preferentially form synapses on VTA GABAergic neurons [9]. Thus, enhanced inhibitory synaptic inputs to VTA-projecting dlBNST neurons should activate VTA GABAergic neurons via a disinhibition mechanism, thereby causing suppression of VTA dopaminergic neurons that may induce depression and anhedonia observed in animal models of chronic pain. In the present study, we hypothesized that inhibitory synaptic inputs to VTA-projecting dlBNST neurons are also enhanced in animal models of depression, thereby suppressing the mesolimbic dopaminergic system. To test this hypothesis, we investigated inhibitory inputs to VTA-projecting dlBNST neurons by whole-cell patch-clamp electrophysiology using brain slices prepared from rats exposed to CMS.

## Materials and methods

**Fig. 1:**
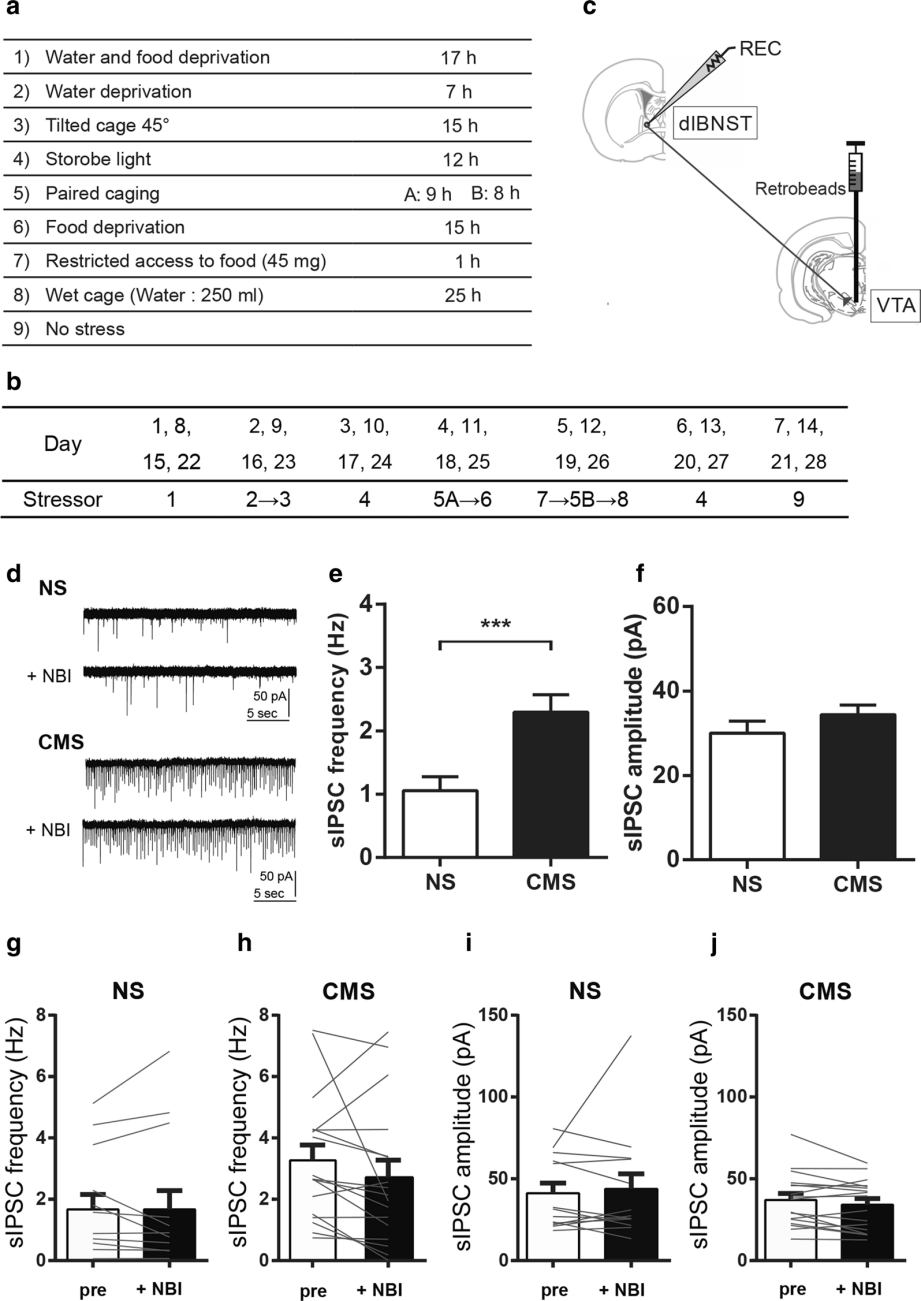
Enhanced sIPSC frequency in VTA-projecting dlBNST neurons in rats exposed to CMS. **a** Stressor protocol. **b** Stress exposure schedule. **c** Schematic diagram of the sites for retrobead injections and patch clamp recordings (REC) from the neurons retrogradely labelled with retrobeads. **d**–**f** Representative traces (**d**), frequency (**e**), and amplitude (**f**) of sIPSCs in VTA-projecting dlBNST type III neurons in the NS and CMS groups. **g**, **h** The effects of NBI27914 on the frequency of sIPSCs in VTA-projecting dlBNST type III neurons in the NS (**g**) and CMS groups (**h**). **i**, **j** The effects of NBI27914 on the amplitude of sIPSCs in VTA-projecting dlBNST type III neurons in the NS (**i**) and CMS groups (**j**). Data are expressed as means ± standard error of the mean. ^***^P < 0.001 (unpaired *t*-test)

Male Sprague-Dawley rats (4 weeks old at the start of stress exposure) were exposed to the eight types of stress shown in Fig. 1a (CMS group) or no stress (NS group) over a 4-week period, according to the schedule shown in Fig. 1b. Electrophysiological experiments were performed within 1 week after the final stress exposure. To visualize VTA-projecting dlBNST neurons, red retrobeads were injected into the VTA (5.5 mm rostral, 1.0 mm lateral, 9.0 mm ventral to bregma) 3–7 days before the electrophysiological experiments (Fig. 1c). The electrophysiological experiments were performed as described previously [8]. Briefly, rats were deeply anesthetized with sodium pentobarbital and transcardially perfused with ice-cold cutting solution. Their brains were quickly removed, and coronal slices (250 µm thick) containing the BNST were prepared in ice-cold cutting solution using a vibratome. The slices were transferred to a submerged recording chamber on an upright microscope and continuously superfused with recording solution at 35 ± 1 °C, saturated with 95% O_2_/5% CO_2_, at a flow rate of 1 ml/min. VTA-projecting dlBNST neurons labeled with retrobeads were visualized using epifluorescence and initially classified into the three types of neurons in current-clamp mode. We previously reported that approximately 80% of VTA-projecting dlBNST neurons are type III [10]. Thus, in this study, spontaneous inhibitory postsynaptic currents (sIPSCs) were recorded from VTA-projecting dlBNST type III neurons, in the presence of 2 mM kynurenic acid to inhibit excitatory postsynaptic currents. In the experiments to examine the effect of NBI27914, a selective corticotropin-releasing factor (CRF) type I receptor antagonist, on sIPSCs, 1 µM NBI27914 was perfused for 15 min, and sIPSCs were analyzed during the 0–3 min before and 12–15 min after the start of the NBI27914 application. The frequency and amplitude of sIPSCs were analyzed using the Mini Analysis Program (Synaptosoft). Data are expressed as means ± standard error of the mean. Statistical analyses were performed using GraphPad Prism version 6. Differences with *P* < 0.05 were considered statistically significant. The detailed materials/methods and datasets used and/or analyzed in this study are provided in Additional files 1, 2, respectively.

## Results

The frequency of sIPSCs in VTA-projecting dlBNST type III neurons was significantly higher in the CMS group (*n* = 41) than in the NS group (*n* = 39) (2.30 ± 0.28 (CMS) vs. 1.06 ± 0.22 (NS) Hz, *t*_78_ = 3.494, *P* = 0.0008, unpaired *t*-test; Fig. 1d, e). The sIPSC amplitude did not significantly differ between the CMS and NS groups (34.41 ± 2.32 (CMS) vs. 30.04 ± 2.88 (NS) pA *t*_78_ = 1.186, *P* = 0.2391, unpaired *t*-test; Fig. 1d, f). There are no differences of cellular properties of recorded neurons between the CMS and NS groups (Additional file 3).

After the recordings of the basal levels of sIPSCs, the effect of NBI27914 on sIPSCs in VTA-projecting dlBNST type III neurons was examined in some neurons (17/41 and 13/39 neurons in the CMS and NS groups, respectively). In the CMS group, bath-application of NBI27914 tended to decrease the frequency and amplitude of sIPSCs, although the effects were not significant (frequency: 3.27 ± 0.50 (pre) to 2.72 ± 0.56 (NBI) Hz, *t*_16_ = 1.415, *P* = 0.1763, paired *t*-test, Fig. 1h; amplitude: 37.12 ± 3.98 (pre) vs. 34.15 ± 3.75 (NBI) pA, *t*_16_ = 1.791, *P* = 0.0922, paired *t*-test, Fig. 1j). In the NS group, NBI27914 did not change the frequency and amplitude of sIPSCs (frequency: 1.67 ± 0.48 (pre) vs. 1.67 ± 0.61 (NBI) Hz, *t*_12_ = 0.007, *P* = 0.9949, paired *t*-test, Fig. 1g; amplitude: 41.18 ± 6.19 (pre) vs. 43.80 ± 9.32 (NBI) pA, *t*_12_ = 0.447, *P* = 0.6629, paired *t*-test, Fig. 1i).

## Discussion

The present study revealed that the frequency of sIPSCs in VTA-projecting dlBNST neurons was enhanced in rats exposed to CMS, an animal model of depression, as observed in our previous study using an animal model of chronic pain [8]. Enhanced inhibitory transmission to VTA-projecting dlBNST neurons may be a neuronal mechanism common to depression and chronic pain that causes depression-like behaviors. Recently, Pati et al*.* reported that the frequency of sIPSCs in dlBNST neurons projecting to the VTA/lateral hypothalamus was enhanced in an animal model of alcohol withdrawal [11]. Enhanced inhibitory transmission to VTA-projecting dlBNST neurons may also be involved in alcohol withdrawal-induced depression.

We previously demonstrated that CRF selectively depolarizes dlBNST type II neurons [12] and increases the inhibitory synaptic inputs to dlBNST type III neurons [13]. Additionally, Marcinkiewcz et al. reported that CRF neurons in the BNST form local GABAergic synapses with BNST neurons that project to the VTA and mediate fear- and anxiety-like behaviors [14]. These findings suggest that at least one of the possible sources of inputs to VTA-projecting dlBNST neurons are dlBNST intrinsic neurons.

We previously reported that enhanced inhibitory synaptic inputs to VTA-projecting dlBNST neurons observed in the animal model of chronic pain were suppressed by NBI27914, a CRF type I receptor antagonist [8]. However, the current study showed that NBI27914 tended to suppress the CMS-induced facilitation of inhibitory synaptic inputs to VTA-projecting dlBNST neurons, but the effects were not significant, suggesting the different neuroplastic mechanisms for the enhancement of inhibitory synaptic inputs to VTA-projecting dlBNST neurons between CMS and chronic pain. Recently, Normandeau et al. reported that neurotensin and CRF co-acted to increase inhibitory synaptic transmission in the dlBNST of non-stressed rats and that CMS bolstered this potentiation through an enhanced contribution of neurotensin over CRF [15]. These findings suggest that neurotensin may be involved in the CMS-induced facilitation of inhibitory synaptic inputs to VTA-projecting dlBNST neurons. Further studies are needed to elucidate what kinds of neurotransmitters and/or neuropeptides are involved in the CMS-induced facilitation of inhibitory synaptic inputs to VTA-projecting dlBNST neurons.

Optogenetic activation of VTA-projecting BNST neuron terminals has been shown to produce rewarding effects in a real-time place preference test [16, 17]. Most VTA-projecting BNST neurons are GABAergic neurons that preferentially form synapses on VTA GABAergic neurons [9]. Thus, activation of VTA-projecting BNST neurons should promote VTA dopaminergic neuron activity by inhibiting the VTA GABAergic neurons that negatively regulate VTA dopaminergic neurons. In this context, enhanced inhibitory synaptic inputs to VTA-projecting BNST neurons may activate VTA GABAergic neurons via a disinhibition mechanism, thereby inhibiting VTA dopaminergic neurons. Suppression of the mesolimbic dopaminergic system may cause anhedonia in animal models of depression and chronic pain.

## Supplementary information

**Additional file 1.** Detailed materials/methods.

**Additional file 2.** Datasets used and/or analyzed during the current study.

**Additional file 3.** Cellular properties of recorded neurons in the NS and CMS groups.

## Data Availability

All data generated or analyzed during this study are included in this published article and its additional information files.

## Abbreviations

BNST: Bed nucleus of the stria terminalis
CMS: Chronic mild stress
CRF: Corticotropin-releasing factor
dlBNST: Dorsolateral bed nucleus of the stria terminalis
NAc: Nucleus accumbens
NS: No stress
sIPSC: Spontaneous inhibitory postsynaptic current
VTA: Ventral tegmental area
