# Supplementary material for: Inhibitory synaptic transmissions to the bed nucleus of the stria terminalis neurons projecting to the ventral tegmental area are enhanced in rats exposed to chronic mild stress
Source: Mol Brain. 2020 Oct 15;13:139. doi: 10.1186/s13041-020-00684-4 (PMC7560094; doi:10.1186/s13041-020-00684-4)
Supplement: Supplementary file 1 — Additional file 1. Detailed materials/methods. [file 13041_2020_684_MOESM1_ESM.pdf]

Additional file 1

Detailed materials/methods

Animals and chronic mild stress (CMS) exposure

Male Sprague–Dawley rats (Japan SLC, Hamamatsu, Japan) were housed in a room with a constant ambient temperature (23 ± 1°C) under a 12-h light/dark cycle, with food and water available *ad libitum*. All experiments were performed with the approval of the Hokkaido University Institutional Animal Care and Use Committee. To prepare the CMS model, rats were exposed to the eight types of stress shown in Fig. 1A (CMS group) or no stress (NS group) over a 4-week period, according to the schedule shown in Fig. 1B. Rats were 4 weeks old at the start of stress exposure. Electrophysiological experiments were performed within 1 week after the final stress exposure.

Electrophysiological experiments

Retrograde tracer (retrobeads) was injected into the VTA 3–7 days before slice preparation. Specifically, rats were fixed in a stereotaxic apparatus (SR-6R-HT; Narishige, Tokyo, Japan) under anesthesia with isoflurane (2%). Topical lidocaine (Aspen Japan K.K., Tokyo, Japan) was administered at incision sites to alleviate pain. Small holes were drilled in the skull, and a 33-gauge Hamilton syringe connected to a microsyringe pump (SYS-MICRO4; World Precision Instruments, Sarasota, FL, USA) was inserted. Rats were unilaterally injected with 0.3–0.4 µl of red retrobeads into the VTA (5.5 mm rostral, 1.0 mm lateral, 9.0 mm ventral to bregma) (Paxinos and Watson, 2007) at a constant rate of 0.075 µl/min and left for an additional 5 min

to prevent backflow. The injection site was checked during slice preparation.

To prepare brain slices, rats were deeply anesthetized with sodium pentobarbital and transcardially perfused with ice-cold cutting solution (in mM: 92 N-methyl-D-glucamine, 2.5 KCl, 30 $NaHCO_3$, 1.25 NaH_2PO_4, 25 glucose, 5 ascorbic acid, 0.5 $CaCl_2$, 20 HEPES, 10 $MgSO_4$, 2 thiourea, 3 sodium pyruvate, and 12 N-acetyl-L-cysteine, oxygenated with 95% O_2/5% CO_2 at pH 7.3 ± 0.1 adjusted with HCl). Their brains were quickly removed, and coronal slices (250 μm thick) containing the BNST were prepared in ice-cold cutting solution using a vibratome (VT1200S; Leica Microsystems GmbH, Wetzlar, Germany). The slices were incubated for 15 min at 30–34°C in cutting solution and subsequently incubated in recording solution (in mM: 119 NaCl, 2.5 KCl, 24 $NaHCO_3$, 1.25 NaH_2PO_4, 12.5 glucose, 2 $CaCl_2$, and 2 $MgSO_4$, oxygenated with 95% O_2/5% CO_2) at room temperature for at least 1 h. The slices were transferred to a submerged recording chamber on an upright microscope (BX50WI; Olympus, Tokyo, Japan) and continuously superfused with recording solution at 35 ± 1°C, saturated with 95% O_2/5% CO_2, at a flow rate of 1 ml/min. Glass pipettes were pulled from thin-walled borosilicate glass capillaries using a micropipette puller (Model P-1000IVF; Sutter Instruments, Novato, CA, USA). Tip resistance was 4.0–8.0 MΩ.

VTA-projecting neurons labeled with retrobeads were visualized using epifluorescence at a 40× objective (LUMPlanLF N 40×/0.80; Olympus). Glass pipettes were filled with a high-chloride internal solution (in mM: 110 KOH, 2 $MgCl_2$, 50 KCl, 0.2 EGTA, 2 Na_2-ATP, 0.3 Na_3-GTP, 10 HEPES, and 0.1 spermine, adjusted to pH 7.3 ± 0.1 with gluconic acid). Because dlBNST neurons have been categorized into three distinct types, VTA-projecting dlBNST

neurons were initially classified into the three types in current-clamp mode. Specifically, these cell types were identified by assessing membrane potential responses to hyperpolarizing and depolarizing current injections. To monitor the responses of dlBNST neurons to hyperpolarizing current injections, the initial membrane potential was adjusted to –60 mV, and a series of currents (–40 pA steps, 400 ms in duration) ranging from 0 to –200 pA were injected. To monitor the responses to depolarizing current injections, the initial membrane potential was adjusted to –80 mV, and a series of currents (+40 pA steps, 400 ms in duration) ranging from 0 to 200 pA were injected. Hyperpolarization-activated cation current (I_h) was identified as described previously [9]. Specifically, the occurrence of I_h was verified by the presence of a "voltage-sag" equal to or larger than 10% of the total voltage deflection upon hyperpolarizing current injection, which elicited a membrane voltage of approximately –100 mV. I_h-positive neurons that exhibited regular firing patterns in response to depolarizing current injections were classified as type I. I_h-positive neurons exhibiting rebound spiking in response to termination of hyperpolarizing current injections and burst spiking in response to depolarizing current injections were classified as type II. I_h-negative neurons were classified as type III.

After classification, the membrane potential was held at –60 mV under voltage-clamp mode, and sIPSCs were recorded. We previously reported that approximately 80% of VTA-projecting dlBNST neurons are type III. Thus, sIPSCs were recorded from VTA-projecting dlBNST type III neurons. Kynurenic acid (Sigma-Aldrich, St. Louis, MO, USA) at a concentration of 2 mM was added to the recording solution to inhibit excitatory postsynaptic currents. In the experiments to examine the effect of NBI27914 (selective CRF type I receptor antagonist;

Tocris Bioscience, Ellisville, MO, USA) on sIPSCs, 1 μM NBI27914 was perfused for 15 min, and sIPSCs were analyzed during the 0–3 min before and 12–15 min after the start of the NBI27914 application. All data were acquired using the Multiclamp 700B amplifier and pClamp10 software (Molecular Devices, Sunnyvale, CA, USA). Data from neurons with a resting membrane potential that was more positive than –40 mV, or from neurons in which the action potential did not overshoot, were excluded from the statistical analyses. The frequency and amplitude of sIPSCs were analyzed using the Mini Analysis Program (Synaptosoft, Fort Lee, NJ, USA).

Statistical analysis

Data are expressed as means ± standard error of the mean. Statistical analyses were performed using GraphPad Prism version 6 (GraphPad Software, San Diego, CA, USA). Differences with $P < 0.05$ were considered statistically significant.
