# Supplementary material for: Inhibitory synaptic transmissions to the bed nucleus of the stria terminalis neurons projecting to the ventral tegmental area are enhanced in rats exposed to chronic mild stress
Source: Mol Brain. 2020 Oct 15;13:139. doi: 10.1186/s13041-020-00684-4 (PMC7560094; doi:10.1186/s13041-020-00684-4)
Supplement: Supplementary file 3 — Additional file 3. Cellular properties of recorded neurons in the NS and CMS groups. [file 13041_2020_684_MOESM3_ESM.pdf]

Additional file 3

Cellular properties of recorded neurons in the NS and CMS groups

	NS			CMS		
Input resistance (MΩ)	726.50	±	76.85	677.99	±	62.69
Resting membrane potential (mV)	-56.21	±	1.36	-56.51	±	1.25
Action potential threshold (mV)	-53.29	±	0.98	-55.83	±	1.12
Time constant (ms)	52.56	±	4.49	52.05	±	3.09
n	39			41		
